# B‐cell haematological malignancies and SARS‐CoV‐2 infection: Could immunological interventions influence the outcome?

**DOI:** 10.1002/jha2.249

**Published:** 2021-06-19

**Authors:** Juliana Ochoa‐Grullón, Ascensión Peña Cortijo, Kissy Guevara‐Hoyer, Carlos Jiménez García, Eduardo de la Fuente, Antonia Rodríguez de la Peña, Miguel Fernández‐Arquero, Ata González Fernández, Silvia Sánchez‐Ramón

**Affiliations:** ^1^ Department of Clinical Immunology and IdISSC Hospital Clínico San Carlos Madrid Spain; ^2^ Department of Immunology Ophthalmology and Otorhinolaryngology (IOO) Complutense University School of Medicine Madrid Spain; ^3^ Department of Haematology Complutense University School of Medicine Madrid Spain

## Abstract

B cell haematological malignancies (HMs) have been described as the worst cancer type for concomitant COVID‐19 in terms of mortality, with rates up to 65%. This risk factor for COVID‐19 cannot only be explained by comorbidities and advanced age of patients, but aggravated by secondary immunodeficiency (SID). We aimed at evaluating the impact of COVID‐19 on 86 HM patients with concomitant SID from a single centre. Only 14 HM patients of 86 (16.28%) patients suffered COVID‐19, with mortality rate of 7%. When we considered patients according to B‐cell defect only or multiple immune defect overlap (B‐T‐cell/NK cells/complement), patients with immune defect overlap presented 5.30‐fold higher risk of COVID‐19 than only B cell defect (95% CI, 1.67–17.0) (*p* = 0.004). Seven (50%) patients were on active IgRT; while five (36%) had received prior mucosal vaccines for respiratory infections. Our results show that modelling SID in HM may contribute to better prediction of infectious risk and to prompt more targeted and timely preventive therapies.

**Abbreviations**
FL: follicular lymphoma
IgRT: immunoglobulin replacement therapy
MGUS: monoclonal gammopathy of unknown significance
NHL: non‐Hodgkin lymphoma
NT: not tested
TIbV: trained immunity‐based vaccine

## 1 INTRODUCTION

According to different studies, case fatality rate for SARS‐CoV‐2 virus infection (COVID‐19) in B cell hematologic malignancy (HM) ranges between 14% and 65% depending on patient's age and need of intensive care unit (ICU) intervention, an extremely high rate considering the 6%–8% mortality among age‐adjusted patients without cancer [1, 2, 3]. Pre‐existing co‐morbidities (older age, chronic lung/heart disease, obesity, diabetes, hypertension) contribute to a more severe course of the disease [4]. In general terms, HM carries an excess risk of infection due to secondary immunodeficiency (SID) that accounts for a significant proportion of mortality, both from underlying malignancy and chemotherapeutic protocols [5]. Numerous publications point to HM to be the worst cancer type for concomitant COVID‐19, probably aggravated by SID [6]. However, neither immunological data to stratify HM infectious risk nor the effects of infectious preventive strategies on COVID‐19 outcome were previously considered. COVID‐19 comprises a broad clinical spectrum ranging from asymptomatic infection to severe multi‐organ failure and death [7].

**FIGURE 1:**
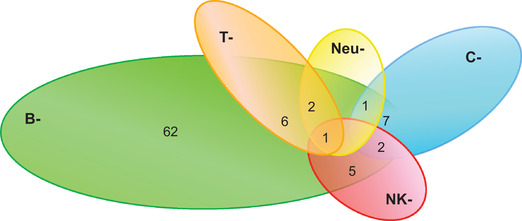
Modelling distribution of SID according to the specific immune defect. Percentage distribution of immune defect: B^‐^ 100% (61), C^‐^ 12% (10), T^‐^ 10% (9), NK^‐^ 9% (8), Neu^‐^ 6% (5)

Assessment of immunological status in HM patients and immunological interventions to prevent infections have shown to reduce bacterial and some viral infections and to improve respiratory prognosis [8, 9, 10, 11, 12]. We aimed at evaluating the impact of COVID‐19 on 86 HM patients with SID from the Hospital Clínico San Carlos of Madrid, Spain during the pandemic era. All 86 patients associated antibody deficiency or memory B cell defect, while 24 of 86 (29%) presented multiple immune defect overlap, such as CD4^+^ T lymphocytopenia and innate immunity alterations (hypocomplementemia, neutropenia or low NK cells counts) (Figure 1). Of the global cohort, 54 (63%) patients were on immunoglobulin replacement therapy (IgRT), and 40 (47%) had received mucosal trained immunity‐based vaccines (TIbV) to prevent recurrent infections [12]. From March, 14 2020 to March, 16 2021, only 14 of 86 (16%) HM patients of our cohort presented with COVID‐19 based on clinical manifestations and real‐time polymerase chain reaction assay. Not expected, COVID‐19 rate was low with favourable outcome, as shown in Table 1. Mean age was 66.35 ± 16.72 years, 50% females. Regarding co‐morbidities, only three (21%) patients had no co‐morbidities, four (29%) patients presented at least one or two comorbidities, and the remaining seven (50%) presented several co‐morbidities (hypertension, COPD, aneurysm, dyslipidaemia, psoriasis, primary biliary cholangitis, chronic kidney disease). Four patients (20%) were asymptomatic and diagnosed following exposure to a confirmed close symptomatic contact who was tested positive for SARS‐CoV‐2; seven patients (50%) had commonly associated COVID‐19 symptoms including anosmia, ageusia, asthenia, myalgias, cough, nasal congestion, dyspnoea, fever and pneumonia; and only three patients (21%) required hospitalization, one of them ICU intervention. None of the patients were on active anticancer therapy. Interestingly, from 14 HM COVID‐19 patients, seven (50%) were on active IgRT, five (36%) had received prior TIbV for respiratory infections, and the remaining two patients were clinically stable, so infection prophylaxis was not considered. Full recovery from COVID‐19 was achieved in 86% (*n* = 12) of the patients, one patient reported persistence of symptoms, particularly asthenia, myalgias and anosmia one year after symptoms onset. One patient passed away due to respiratory complications during hospitalization. This patient suffered an underlying chronic kidney disease, associated a combined T‐B immunodeficiency. After COVID‐19, nine of 14 patients were tested for anti‐SARS‐CoV2 antibodies, and seven of them (78%) tested positive.

**TABLE 1:** Baseline demographic and immunological characteristics of patients with B cell haematological malignancies and SARS‐CoV‐2 infection

Patient	Diagnosis HM	Age (years)	Sex	Clinical symptoms	Duration (days)	ICU	Outcome	Anti‐SARS‐CoV2 Ab	Immunological characteristics	IgRT	TIbV
*#7*	CLL	65	F	Fever 38 °C, headache, myalgias	8	No	Recovery	Yes	Antibody deficiency	Yes	No
*#8*	CLL	71	F	Headache	6	No	Recovery	NT	Antibody deficiency + C4 hypocomplementemia	No	Yes
*#26*	NHL (FL)	74	F	Fever, pneumonia, asthenia	2	No	Exitus	Yes	Antibody deficiency, marked CD4+ T‐lymphocytopenia and neutropenia	Yes	No
*#29*	NHL (FL)	76	F	Asymptomatic	0	No	Recovery	Yes	Antibody deficiency and low NK cells	No	Yes
*#33*	NHL (FL)	55	M	Asymptomatic	0	No	Recovery	Yes	Antibody deficiency	No	Yes
*#37*	NHL (FL)	77	F	Asymptomatic	0	No	Recovery	Yes	Antibody deficiency and C4 hypocomplementemia	No	No
*#40*	NHL (FL)	58	F	Cough, nasal congestion, headache	10	No	Recovery	NT	Antibody deficiency	No	Yes
*#47*	NHL (DLBCL)	82	M	Chest pain	2	No	Recovery	No	Antibody deficiency and CD4+ T‐lymphocytopenia	Yes	No
*#54*	HL	27	M	Anosmia, ageusia	12	No	Recovery	NT	Antibody deficiency and CD4+ T‐lymphocytopenia	Yes	Yes
*#62*	MGUS/MM IgA Lambda	75	F	Fever, pneumonia, asthenia	21	Yes	Recovery	Yes	Antibody deficiency	No	No
*#65*	MGUS IgG K	49	M	Fever, pneumonia, asthenia, anosmia	7	No	Persistence of anosmia, myalgias and asthenia	NT	Antibody deficiency	No	No
*#74*	MGUS IgG K	51	M	Fever, respiratory distress	6	No	Recovery	No	Antibody deficiency, C4 hypocomplementemia and low NK cells	No	No
*#78*	Waldenström disease	78	M	Asymptomatic	0	No	Recovery	Yes	Antibody deficiency	No	No
*#86*	Monoclonal B lymphocytosis	91	M	Pneumonia	8	No	Recovery	NT	Antibody deficiency	Yes	No

CLL: chronic lymphocytic leukemia; FL: follicular lymphoma; HL: Hodgkin lymphoma; MGUS: monoclonal gammopathy of unknown significance; NHL: non‐Hodgkin lymphoma.

Age above 60 has been defined as the most important single aspect associated with high mortality. In our cohort, 64% (*n* = 9) were older than 60 years with mortality rate of 11%, a much lower frequency than that published so far (47%) in HM population [1]. Despite the small size of our cohort, global mortality rate was 7% (1/14), a figure comparable for the control population (6%–8%). HM patients under active IgRT, which was the majority of them, continued to attend the hospital day care monthly during the pandemic, while only 7 of 54 (13%) presented COVID‐19 so far. Although B cell defect is the earliest and commonest defect in patients with HM, a significant proportion of HM patients present overlap of multiple innate and adaptive immune defects. We sought to analyse whether the risk of COVID‐19 was different between ‘B cell defect’ and ‘immune defect overlap’. Interestingly, ‘immune defect overlap’ was associated with significantly higher risk of SARS‐CoV2 infection than ‘B cell defect’ (OR, 5.30; 95% CI, 1.67–17.0) (*p* = 0.004). The patients that presented an ‘immune defect overlap’ associated a 3.24‐fold higher risk for prior severe infection (*n* = 21; 24%) (95% CI, 1.14–9.19) with respect to patients with ‘B cell defect’ (*p* = 0.02). After initiating preventive immune‐based strategies (IVIg/TIbV), only three patients during a follow‐up of 36 months had occurrence of severe infection, two of them with immune defect overlap and one patient with B cell defect and chronic renal failure who suffered from severe COVID‐19.

Globally, immunological work‐up and infectious preventive interventions in our cohort of SID HM patients might explain the positive COVID‐19 evolution beyond other factors. Immune response to SARS‐CoV‐2 relies primarily on innate immunity [13], which has been shown to be enhanced by TIbV. This mucosal preparation is composed by mixed Gram negative and positive inactivated bacteria that synergistically activate innate immunity through TLR sensors and favour specific adaptive responses (Benito‐Villabilla 2017). On the other hand, a protective effect of IgRT against COVID‐19 is yet to be proven, although previous studies of SARS and Middle East respiratory syndrome have exhibited clinical benefits of IVIg therapy [14]. IVIg seems to contain cross‐reactive antibodies against SARS‐CoV‐2 due to common circulating coronaviruses [15]. The first plasma pool of IVIg that tested positive for anti‐SARS‐CoV‐2 antibodies in Spain was in July 2020 [16], and all infected patients were diagnosed during the first wave before July. Immunomodulatory effects of IVIg both on innate and adaptive immunity might also have played a role [17, 18].

We agree, as Vijenthira et al claim, in the urgent necessity to collect data on selected HM patients’ population to ascertain the true risk of COVID‐19 mortality. It seems reasonable to stratify risk through immunological assessment. Our results suggest that SID classification predict COVID‐19 risk and that targeted preventive strategies in SID HM patients might be beneficial to mitigate potential complications in this life‐threatening COVID‐19 group. Further studies are necessary to validate these preliminary results.

## CONFLICT OF INTEREST

The authors declare that the research was conducted in the absence of any commercial or financial relationships that could be construed as a potential conflict of interest.

## AUTHOR CONTRIBUTIONS

J. Ochoa‐Grullón, K. Guevara‐Hoyer and S. Sánchez‐Ramón contributed to conception and design of the study. E. de la Fuente and C. Jiménez García contributed to the database. J. Ochoa‐Grullón wrote the first draft of the manuscript. A. R. de la Peña has contributed to the immunological study of SID. S. Sánchez‐Ramón wrote sections of the manuscript and reviewed the manuscript. All authors contributed to the article and approved the submitted version.

### FUNDING INFORMATION

This work was supported by CRIS Cancer Foundation (SSR.C01CRIS) and Caja Sur.

### INSTITUTIONAL REVIEW BOARD STATEMENT

The study was conducted according to the guidelines of the Declaration of Helsinki and approved by the Institutional Review Board. The study was approved by the Ethics Committee of the Hospital Clínico San Carlos (20/243‐E_BS).

## Data Availability

The raw data supporting the conclusions of this article will be made available by the authors, without undue reservation.
